# Evaluation of Continuity of Care: What Can Physician Survey Add?

**DOI:** 10.5334/ijic.7018

**Published:** 2024-02-01

**Authors:** Igor Sheiman, Sergey Shishkin

**Affiliations:** 1 National Research University High School of Economics (Moscow), Russian Federation; 2 National Research University High School of Economics, Russian Federation

**Keywords:** care integration, continuity of care, evaluation of integration, physician survey, Russian Federation

## Abstract

**Background:**

The evaluation of continuity of care is usually based on the indicators of the frequency of patients’ contacts with specific providers. There are some first attempts to use physician survey for the evaluation.

**Objective:**

Is to get additional information on the continuity of care in Russia by a newly developed physician questionnaire with detailed questions related to the specific areas of providers’ interaction in the health system.

**Methods:**

The questionnaire was developed to increase the number of characteristics and indicators for the evaluation of informational, longitudinal and interpersonal continuity. Each of 17 questions was pretested by a group of experts. A small physician survey was conducted through the mobile App with 2690 respondents. A sample is skewed to young and urban respondents. The attempts have been made to increase its representativeness.

**Results and discussion:**

We identified the areas of low continuity of care in Russia. Access to electronic medical records is limited. Outpatient and inpatient physicians rarely contact with each other. Primary care physicians are unaware of the substantial part of hospital admissions and emergency visits of their patients, which makes them unprepared for the follow-up treatment. Home visits to patients with heart attack and stroke after hospital discharge are rare. The lack of timely transfer of hospital cases to rehabilitative and social care settings also limits continuity of care. However, a small scale of the survey and its online operation limit its representativeness and robustness. Bigger scale of the survey with the same or similar questionnaire can improve its results.

**Conclusion:**

Physician survey can be a useful instrument of care continuity evaluation. The content of the suggested survey can be valuable for collecting the international evidence.

## Introduction

Strengthening continuity of care is critical to achieve its high outcomes. A growing number of people with chronic diseases and long-term conditions require not only more diverse and complex medical interventions but also a close interaction of care providers to ensure a smooth progress of patients in the health system [1]. According to Haggerty et al. [2], “continuity is the degree to which a series of discrete health care events is experienced as coherent and connected, and is consistent with the patient’s medical needs and personal context”. It means that health care is not limited to one contact with health provider and one episode of care but relates to patients needs longitudinally and at various stages of service delivery. Continuity is a major characteristic of care integration, together with teamwork and coordination of care [3]. Moreover, it is a major focus of strengthening integration: “coordination and teamwork is what providers do for the benefit of continuity” [4]

There is a consensus on three types of continuity. Informational continuity means that a provider has an access to all relevant information about patients’ utilization of care at all stages of their “route” in the health system. Longitudinal continuity means that it transcends multiple episodes of disease, and interpersonal relates to a trustful relationship of patients and providers [5]. Sometimes interpersonal continuity is used as a proxy measure for the strength of patient-physician relationships [6].

A substantial body of literature addresses the impact of continuity on health systems performance. There is an evidence that a frequency of contacts with one doctor is a strong factor of patient satisfaction, improved health promotion [7,8]. Many studies report reductions in mortality, with increased interpersonal continuity of care. A well-organized post-discharge care at home decreases utilization of inpatient care and mortality [9].

Another body of literature focuses on evaluating continuity. It addresses mostly the interpersonal continuity and measures the frequency of patient contacts with providers using Continuity of Care Index, Usual Provider Continuity Index, etc. [10,11]. These studies are based on the statistical data, patient assignment data systems, surveys of patients. But such measurement technique has some limitations. Quantitative indexes don’t characterize fully the complex nature of interactions between providers and patients, as well as between providers (longitudinal and informational continuity). Many low and medium income countries do not have accurate patient assignment data systems, while statistical data is often not available to the public.

The recent OECD study [12] has contributed to the measurement of integration by suggesting three indicators with the focus on care continuity: mortality, hospital readmissions and prescribed medicines for post-discharge care in stroke and congestive heart failure patients. The measurement was based on patient-level data across various datasets. This study covered 15 countries and provided useful results in collection of comparative data. The Commonwealth Fund study [13] is another attempt to compare integration in 11 OECD countries. The survey queried primary health care physicians about their ability to coordinate patients’ medical care with specialists, across settings of care, and with social service providers. The survey was based on the nationally representative samples of physicians.

The objective of this paper is to get additional information on the continuity of care in Russia by a newly developed physician questionnaire. Contrary to the international studies, it is designed to evaluate a substantial number of characteristics and indicators of informational, longitudinal and interpersonal continuity of care. We want to show what specifically can be assessed with physician survey, as well as the limitations of this evaluation instrument in the context of the Russian Federation and internationally.

Following is a summary of the specific characteristics of care organization in Russia related to the evaluation of care continuity: 1) Most of primary and outpatient specialty care is provided in multi-specialty polyclinics. The interaction of generalists and outpatient specialists exists “under one roof”. The referrals for outpatient care to external entities are relatively rare. This facilitates but does not guarantee teamwork and continuity of care [14]. 2) The hospital sector is built as a multilevel system, with rural, central rayon, city, regional and federal hospitals closely linked through a referral system from one level to another, while outpatient and inpatient care is usually provided by different doctors [15]. 3) The distinction between acute and long-term hospitals does not exist in Russia. Nursing homes and similar post-acute institutions are rare [15]. The links between the two are not clear. 4) Emergency care is provided by stand-alone entities – stations of medical emergency. They are supposed to communicate with polyclinics and hospitals. Emergency care units of hospitals, typical for many Western countries, are rare in Russia. 5) Social care is developing as a separate sector. Polyclinics and hospitals can refer their patient to social facilities, but there are many barriers to their joint activities [14]. 6) There is a sector of spas, where patients can be referred by polyclinics for rehabilitation. However, a chronic underfunding of health care (public health funding is only 3.5% of GDP) limits the development of this sector and its links with polyclinics; 7) Service delivery is increasingly influenced by electronic medical cards. But the scale of this process is still limited [15].

Bearing in mind some integrative features of health care, Russia is a good case for the evaluation of care continuity. The major research question is how physician survey can contribute to this evaluation. The first attempt was made in 2014 [3], while it covered a limited number of continuity characteristics, and the questionnaire had only four questions on this subject. This paper is a continuation of the study.

## Methods and data

We followed a three-step methodological framework: 1) the choice of major characteristics of three types of continuity (informational, longitudinal and interpersonal) for their evaluation; 2) developing questions for each characteristic; 3) designing a physician survey.

### Major characteristics of continuity for evaluation

#### Informational continuity

Informational continuity implies availability of an organized collection of medical and social information about each patient and its accessibility to any health care professional caring for the patient [6]. We highlighted the following major characteristics of this availability for the evaluation with the use of physicians’ survey.

1. Access of physicians to comprehensive electronic medical records to track the progress of patients in the health system. This is an indication of the capacity for clinical information exchange.
2. Primary care physicians’ awareness of hospital admissions and emergency visits of their enrolled population. The lack of such information is a sign of poor communication links between respective providers.
3. Frequency of clinical information exchange between professionals of various medical settings and units – before, during and after hospital admission. This exchange is particularly important for patients with complex health problems.
4. Frequency of specialists’ feedback to referring general practitioners (GPs). This feedback is a condition for a constant management of patients by their regular physician or coordinator.

#### Longitudinal continuity

Longitudinal continuity refers to an ongoing pattern of health care providers’ interaction and implies availability of the organizational setting in which care can occur and should make it easier for patients to access care when needed [6]. Using physicians’ survey it is possible to evaluate the following characteristics:

1. Use of unobstructed patients’ management technologies to ensure their smooth progress in the health system. The most important is the availability of hospital discharge planning with the aim to ensure timely and appropriate care after the completion of inpatient care episode. This is particularly relevant for “catastrophic” cases of stroke and heart attack.
2. Frequency of hospital re-admissions as an indication of an inappropriate patient management after hospital discharge.
3. Regular clinical and informational links between providers of outpatient, inpatient, rehabilitative and social care. Their lack usually results in poor continuity of care and places an excessive burden on hospitals.

#### Interpersonal continuity

Interpersonal continuity refers to ongoing personal relationship between the patient and care provider, and is characterized by personal trust and responsibility [6]. It includes the following characteristics:

1. Comprehensiveness of care provided by GPs. The degree of interpersonal trust and responsibility is usually higher when a GP provides a higher package of services and the care episode is finished at this level, with no referrals to specialists. The share of primary visits to district physicians that are finished with the referral to outpatient specialists may be used as an indicator of the capacity of PHC physicians to diagnose diseases and treat patients without involvement of specialists. This makes their contacts with patients more stable and implies interpersonal continuity of care.
2. Availability of well-structured chronic disease management activities. A set of these activities is designed for a specific group of patients and includes constant contacts with health professionals [14]. In Russia, these activities are known as “schools of patients” for the specific chronic cases. So the availability of schools of chronic patients may serve as an indicator of the strength of patient-physician relationships.
3. Frequency of primary health care (PHC) physicians’ initiatives to involve social workers to the management of patients with substantial health problems. This is also an indication of close links between patients and physicians.

### Developing the questionnaire

We use a set of questions that follow the characteristics of care continuity. They are further specified for the following interactions between providers: a) district physicians (generalists) and outpatient specialists in the staff of polyclinics; b) polyclinics and hospitals; c) polyclinics and emergency care entities; d) polyclinics, hospitals, rehabilitative and social care entities. These are the major areas where barriers may exist for patients’ movement. Depending on their focus, the questions are addressed to all physicians, polyclinic physicians, hospital doctors. The selection of the respondents subgroups is determined solely by their ability to answer a specific question. For example, hospital doctors can’t estimate interpersonal contacts between primary care physicians and patients, therefore we address the respective questions to polyclinic physicians.

Rural health care in Russia has specific characteristics in terms of the structure of providers, the specific “routes’ of patients flow between providers, much lower level of communication of physicians than in urban areas (partly due to a low density of medical facilities). These distinctions required a set of specific questions addressed to rural physicians. To avoid an excessive respondent burden, we excluded these questions from the survey.

A preliminary questionnaire was developed by the authors and then was reviewed by service delivery experts from the National Research University Higher School of Economics and randomly selected senior health managers from health authorities – 9 experts altogether. The criteria for the review included:

1. validity of each question for the characteristics of continuity;
2. comprehensibility;
3. logical flow;
4. clarity for respondents;
5. possibility of bias due to the inappropriate question wording;
6. possibility of misinterpretation due to the COVID-2919 pandemic;
7. the appropriateness of the suggested intervals for responses;
8. probability of responding (respondent burden).

Most of these criteria are used in the international practice of the development of a questionnaire – according to the guidelines for survey development and reporting guidelines (https://www.equator-network.org/). Also, we asked experts to suggest their own versions of questions and to add new ones.

The responses were not weighted, since the reliability of each criterion was hard to compare. Instead, the responses were discussed in three online and a face-to-face meetings. Some initial questions were declined by all participants – mostly due the low clarity and possible misinterpretation. Most of discussion was focused on the clear specification of questions. The number of the suggested additional questions has doubled their initial list. We concluded that the respondent burden would be too heavy for the online study of continuity, and made a joint decision to start with the most important questions – see Table 1.

**Table 1:** Types of continuity of care and related questions of the physicians’ survey.


TYPES/CHARACTERISTICS OF CONTINUITY	RELATED QUESTIONS	WHOM ARE ADDRESSED

**Informational continuity**		

1. Access to comprehensive electronic medical records	Do you have an access to electronic medical records made in your region?	All physicians questioned

2. Primary care physicians’ awareness of hospital admissions and emergency visits of their enrolled population.	How often does your polyclinic receive information about hospital admissions of patients enrolled with it?	Polyclinic physicians

	How often does your polyclinic receive information about emergency visits of patients enrolled with it?	Polyclinic physicians

3. Frequency of clinical information exchange between professionals	Do you contact district physicians and other outpatient physicians when a patient is admitted and in the process of inpatient care?	Hospital physicians

	Do you contact hospital doctors about clarifications of post-discharge treatment?	Polyclinic physicians

	How often do hospital doctors consult polyclinic physicians on managing patients after hospital admission?	Polyclinic physicians

4. Frequency of specialists’ feedback to referring GPs	How often do you receive a feedback from rehabilitative facilities about the results of care of the patients whom you referred?	Polyclinic physicians

**Longitudinal continuity**		

1. Use of unobstructed patients’ management technologies	Does your polyclinic practice home visits to patients with stroke and heart attack the first days after their hospital discharge?	Polyclinic physicians

2. Frequency of hospital re-admissions	How often are patients re-admitted due to inappropriate management by polyclinics physicians?	Hospital physicians

3. Regular information exchange and clinical links between providers	How often are patients of your hospital transferred to rehabilitative inpatient care entities for the continuation of inpatient care (when it is necessary for a patient)?	Hospital physicians

	How often are patients of your hospital transferred to long-term social care entities (when it is necessary for a patient)?	Hospital physicians

	What is your estimate of the share of hospital beds occupied by patients who need a transfer to the rehabilitative and social entities?	Hospital physicians

	What is your estimate of the degree of continuity of care provided in hospitals and polyclinics?	All physicians questioned

**Interpersonal continuity**		

1. Comprehensiveness of care provided by GPs.	What is your estimate of the share of primary visits to district physicians that are finished with the referral to outpatient specialists?	Polyclinic physicians

2. Availability of well-structured chronic disease management activities.	Is constant chronic patients’ management practiced in your polyclinic?	Polyclinic physicians

3. Frequency of PHC physicians’ initiatives to involve social workers to the management of patients	Do you approach social care providers with the request to help your patients?	Polyclinic physicians


To eliminate the impact of the pandemic, we added to each question “in regular conditions of work before March 2020”. A further analysis of the survey results indicated that respondents had understood this remark and described a usual situation in the health system.

All questions are formulated in the form of suggested responses ranging from three to seven options. Some responses include quantitative intervals. The scales for each question are determined by health data experts based on the estimate of respondents’ willingness and capacity to evaluate options quantitatively. Therefore, the scales differ across questions. The alternative approach is to suggest general options – “always”, “seldom”, “often”, “never” or the option “don’t know”. In both cases, the distribution of responses was estimated.

### Survey design

The evidence on the continuity of care in Russia is based on the physician survey that was conducted online in October 2020 through the mobile App Handbook of Physician (available in Google Play and AppStore https://medsolutions.ru/#/manual) with 540 thousand registered users or 76 % of the total number of physicians in the country. The App provides information on clinical recommendations, new medical technologies and other medical issues. It is operated by the company that contracts research organizations for conducting physician surveys – in addition to its major informational mission.

In our contract with the company, we specified the representation of all regions of the country, the minimum number of respondents (2600 registered users), and asked to provide the distribution across types of medical facilities (polyclinics, hospitals, emergency care stations, other settings), professional groups (general practitioners, district therapists, outpatient specialists, hospital doctors, health managers), and age groups. The company addressed the registered users in all regions of the country with requests to respond to the questionnaire. It repeated requests so that to reach a contracted minimum number of respondents and thereby meet a contracted cost of the survey.

The cycle of communication ranged across regions from 7 to 14 days. The actual number of respondents was close to the specified minimum – 2590 registered users. This is less than one per cent of the total number of physicians in the country and the number of registered users of the App. The respondents represented 81 of 85 regions of the country. Two biggest cities were over-represented – Moscow (392 respondents) and Saint-Petersburg (165). The number of respondents in other regions ranged from 10 to 80 physicians. Given relatively high health funding and more active efforts to streamline organization of care in the biggest cities, their over-representation leads to some overestimation of continuity of care.

1118 respondents work in polyclinics (48%), 1068 – in hospitals (46%), the rest – in other settings. This distribution represents the actual structure of physicians across types of medical facilities in the country.

The professional structure of respondents of polyclinics is skewed to primary care physicians, with 625 generalists, 378 outpatient specialists, 115 managers of polyclinics and their units. The actual structure of polyclinic physicians in the country is different: around two thirds of polyclinic physicians are specialists [16]. The specialty structure of hospital respondents was not evaluated.

The age distribution of respondents: 22–29 years – 22.9%, 30-39 – 32.4%, 40–49 – 19.3%, 50–59 – 183%, 60 years and more – 7.2%. The statistical data on the actual age structure of physicians in the country is unavailable. Intuitionally, young physicians are over-represented in the survey. The country faces a serious problem of a growing number of young physicians leaving clinical work. The health system is aging, with a high proportion of physicians older than 60 years [17].

To overcome all possible limitations of the sample, we used the following instruments:

1. negotiating the rate of the minimum number of respondents and its cost with the operator of the survey;
2. monitoring the preliminary results of the survey across groups of respondents and geographical areas;
3. sending repeated invitations to the under-represented groups of registered users to participate in the survey.

## Results of the survey

Below is the summary of findings for each type of care continuity. We estimate the percentage of responses to the questions by all respondents or their specific group – hospital doctors or polyclinic physicians. The latter include polyclinic managers (they are always physicians). The references are made to the respective figures with the detailed distribution of physicians’ responses to the questions that are given in the appendix (supplementary materials). The absolute numbers can be obtained through the communication with the authors.

### Informational continuity

**Figure 1:**
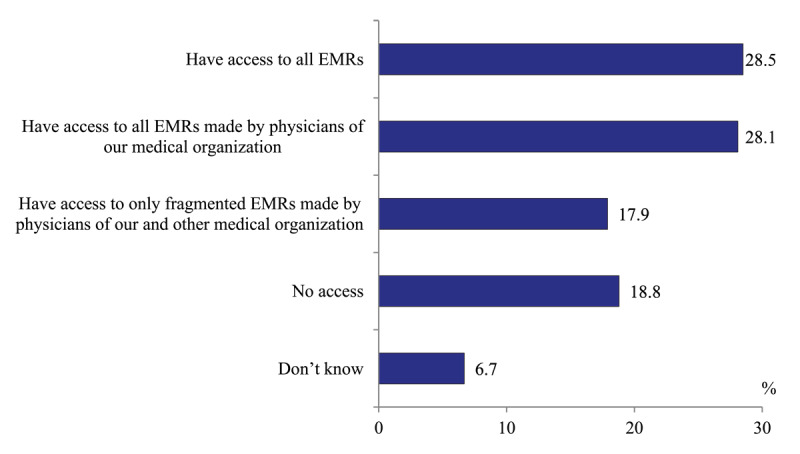
Distribution of polyclinics physicians’ responses to the question “Do you have an access to electronic medical records of patients (tests, consultations, admissions, visits, clinical data), if they were made in your region” in 2020, %.

*Access to comprehensive electronic medical records*. Only 28.5% of polyclinic physicians report that all electronic medical records made in their region are accessible. 28.1% have an access to all records made only in their facility, 17.9 – to some fragmented records, while 18.8% of responses don’t have an access to any electronic records (Figure 1).

**Figure 2:**
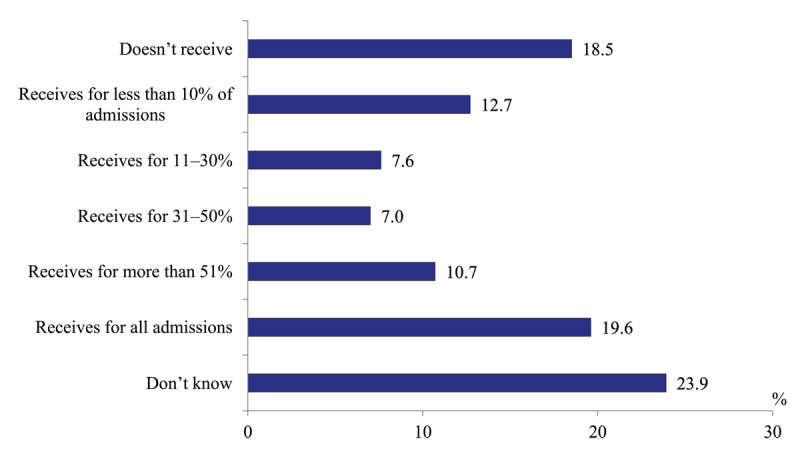
Distribution of polyclinics physicians’ responses to the question “How often does your polyclinic receive information about hospital admissions of patients enrolled with it” in 2020, %.

*Level of polyclinic physicians’ awareness of the current hospital admissions of their patients*. Only 19.6% of polyclinic physicians report that their polyclinic receives information about all admissions and 10.7% – about more than 50% of admissions. 28% of respondents have a fragmented information and 18.5% don’t have any (Figure 2). If to assume that 23.9% of respondents who find it difficult to answer the question are close to the group that gives the answer “Don’t receive at all”, then we can conclude that the level of informing polyclinic physicians is low: they receive information about the hospitalization of their patients approximately only in every fourth case.

**Figure 3:**
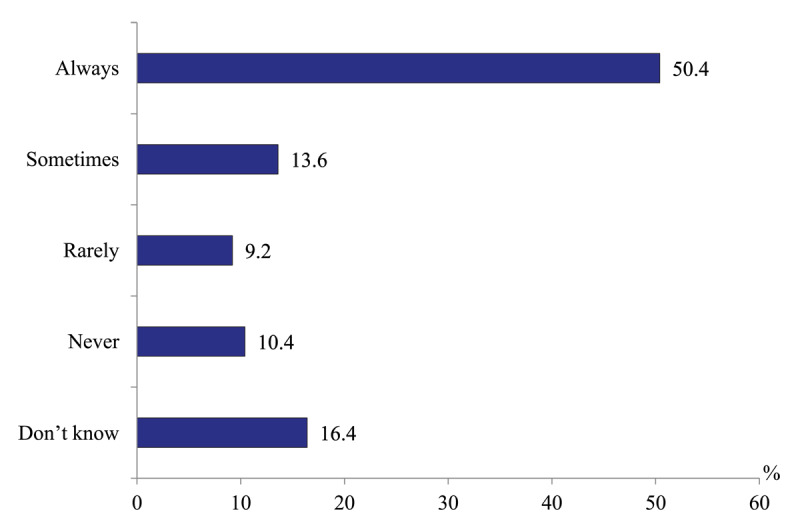
Distribution of polyclinics physicians’ responses to the question “How often does your polyclinic receive information about emergency visits of patients enrolled with it?” in 2020, %.

*Level of polyclinic physicians’ awareness of the current contacts of their patient with organizations (units) of emergency care*. Half (50.4%) of physicians report that their polyclinics are notified of all emergency contacts of their patients. The share of those who are not informed at all is only around 10% (Figure 3).

**Figure 4:**
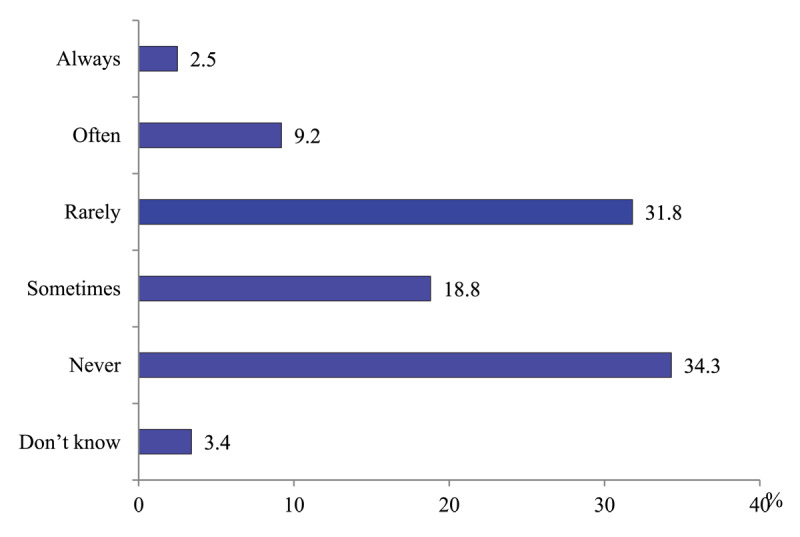
Distribution of hospital physicians’ responses to the question “Do you contact polyclinics physicians when their patients are admitted and treated in a hospital?” in 2020, %.

*Hospital physicians’ contacts with polyclinic physicians*. The question of the survey is focused on the contacts before admission and in the course of inpatient care. More than third of hospital physicians (34.3%) never contact their polyclinic counterparts, 50.6% contact “rarely’ or “sometimes”. The share of those who do this “always” and “often” is 11.7% (Figure 4).

**Figure 5:**
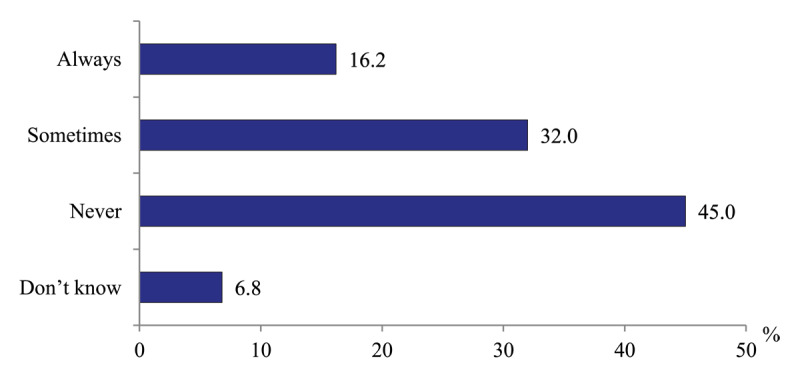
Distribution of polyclinics physicians’ responses to the question “Do you contact hospital physicians about clarifications of post-discharge treatment?” in 2020, %.

*Frequency of consulting polyclinic physicians by hospital doctors on managing patients after hospital discharge*. The survey indicates that 38.2% of polyclinic physicians don’t have any consultations and 33.1% have them “rarely”, while only 14.2% often use this opportunity. Similar question addressed to hospital doctors gives more optimistic estimates: 22.7% – “often”, 40.8% – “rarely” (Figure 5).

**Figure 6:**
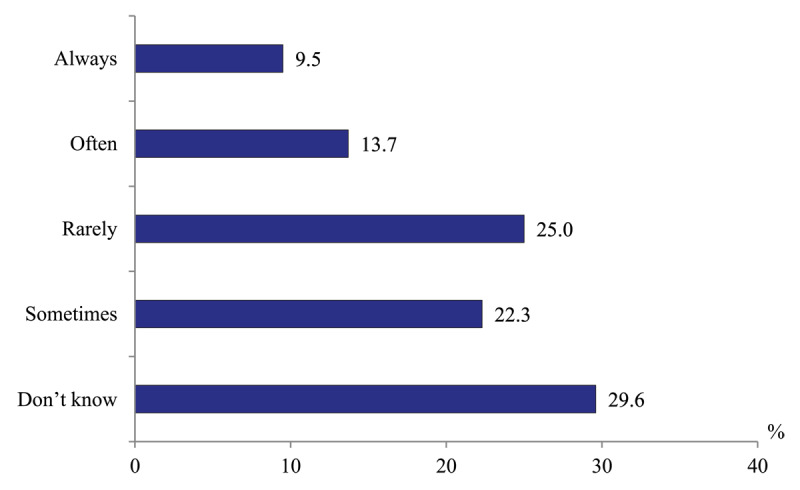
Distribution of polyclinics physicians’ responses to the question “How often do you receive information about results of care in the spa of patients whom you referred?” in 2020, %.

*Frequency of the feedback from spas to a referring physician on the results of treatment*. Only 23.2% of polyclinic physicians report that receive this information “always” or “sometimes”, while 47.3% – “never” or “rarely” (Figure 6).

### Longitudinal continuity

**Figure 7:**
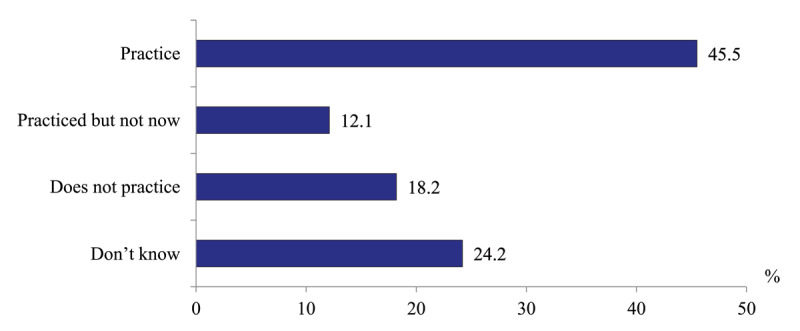
Distribution of polyclinics physicians’ responses to the question “Does your polyclinic practice home visits to patients with stroke and heart attack the first days after their hospital discharge?” in 2020, %.

*Use of unobstructed patients’ management technologies*. Polyclinic physicians are questioned about the availability of the practice of home visits to the patients with stroke and heart attack the first days after discharge. 45.5% of physicians report positively without clarification how often, while the rest does not confirm such visits (Figure 7). These responses relate to physicians visits. The visits of nurses to such patients are practiced rarely because of their limited clinical capacity [16].

**Figure 8:**
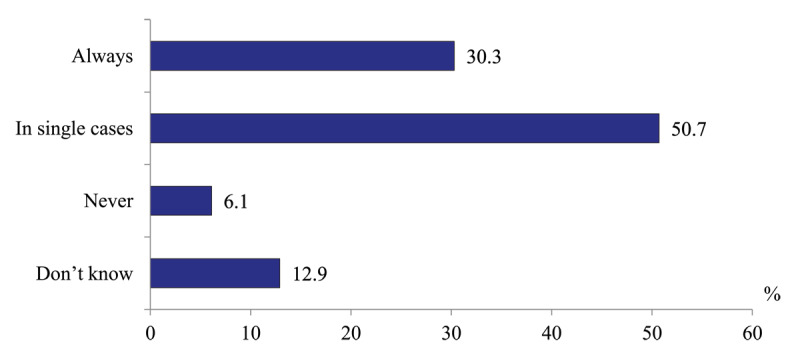
Distribution of hospital physicians’ responses to the question “How often are your patients resubmitted due to inappropriate management by polyclinics physicians?” in 2020, %.

*Level of hospital readmissions due to inappropriate management of patients by polyclinic physicians*. 30.3% of hospital doctors report that they run into this all the time, while 50.7% – “in single cases”. Only 6.1% never face such cases (Figure 8).

**Figure 9:**
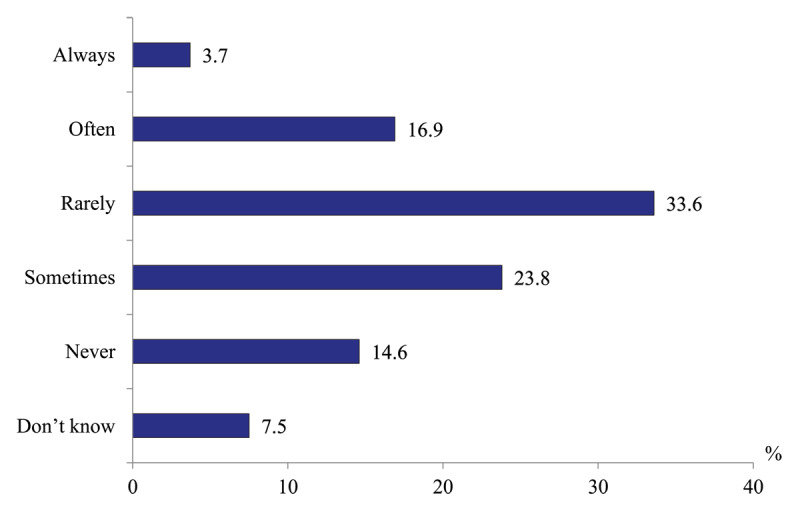
Distribution of hospital physicians’ responses to the question “How often are patients of your hospital transferred to rehabilitative inpatient care entities or units for the continuation of inpatient care (when it is necessary for a patient)?” in 2020, %.

**Figure 10:**
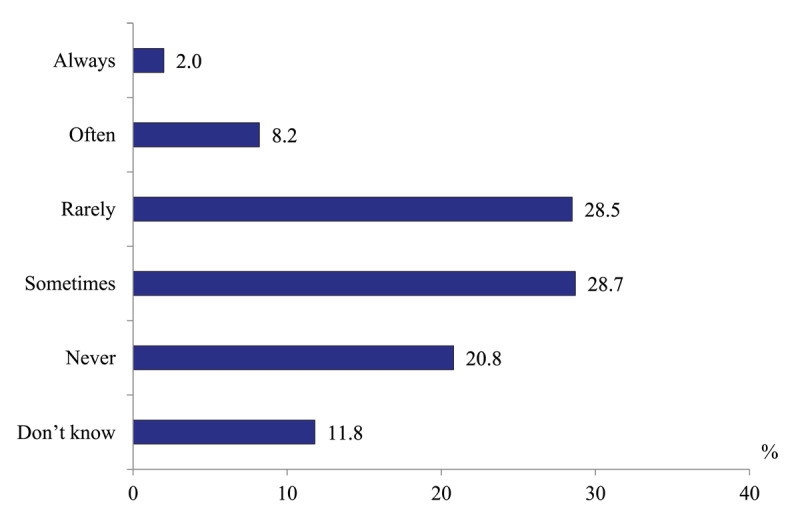
Distribution of hospital physicians’ responses to the question “How often are patients of your hospital transferred to social care entities or units for the continuation of inpatient care (when it is necessary for a patient)?” in 2020, %.

*Frequency of timely transfers of patients from acute hospitals to rehabilitative care and long-term social care facilities*. Only 20.6% of hospital doctors report that their patients can “always” or ‘often” be transferred to rehabilitative inpatient units, while 57.4% report that such transfers rarely happen and 14.6% never transfer their patients to such facilities (Figure 9). Similarly low is the frequency of patients’ transfers to long-term social care institutions (Figure 10).

**Figure 11:**
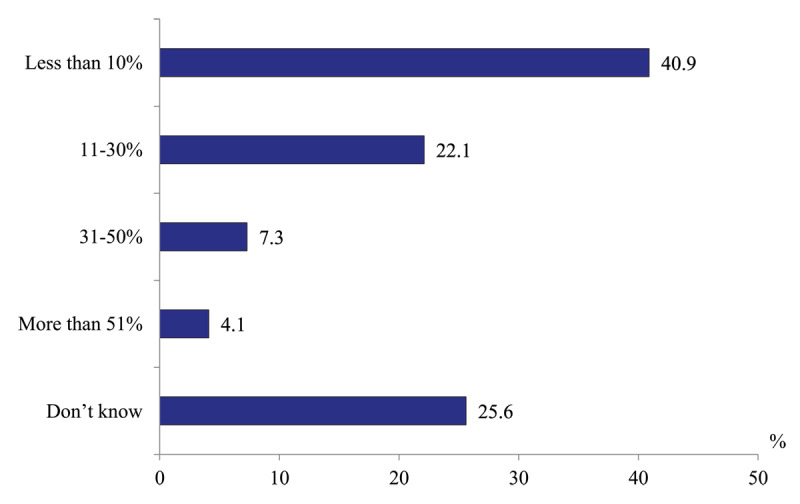
Distribution of hospital physicians’ responses to the question “What is your estimate of the share of hospital beds occupied by patients who need a transfer to the rehabilitative and social entities?” in 2020, %.

*Share of acute hospital beds occupied by patients who need a transfer to the rehabilitative and long-term care entities*. A low percentage is an indication of a smooth progress of patient in the health system. This is not the case in Russia. 33.5% of hospital doctors report that this share is higher than 10% (22.1% – from 11 to 30% of hospital beds). 40.9% of respondents make a more optimistic estimate of less than 10% of beds (Figure 11).

**Figure 12:**
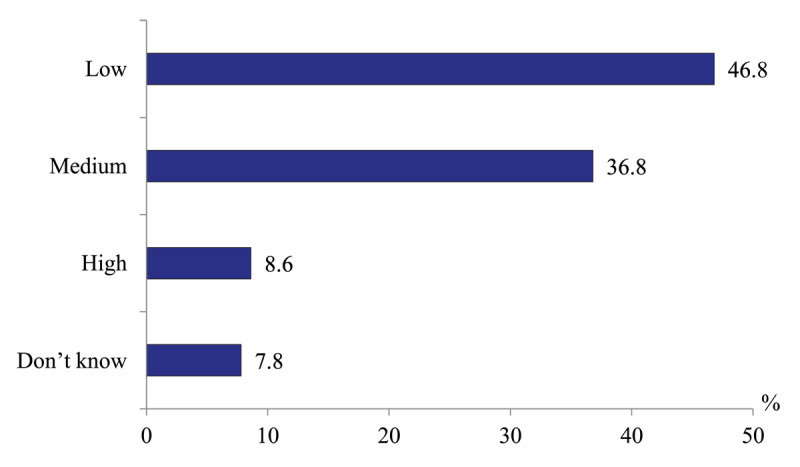
Distribution of all physicians’ responses to the question “What is your estimate of the degree of continuity of care provided in hospitals and polyclinics?” in 2020, %.

*Physicians’ satisfaction with the degree of continuity of care provided in various types of medical facilities*. The opinions of all physicians questioned on the level of continuity of care in their regions are divided: only 8.6% consider it “high”, 36.8% – “medium”, and almost half (46.8%) – “low” (Figure 12).

### Interpersonal continuity

**Figure 13:**
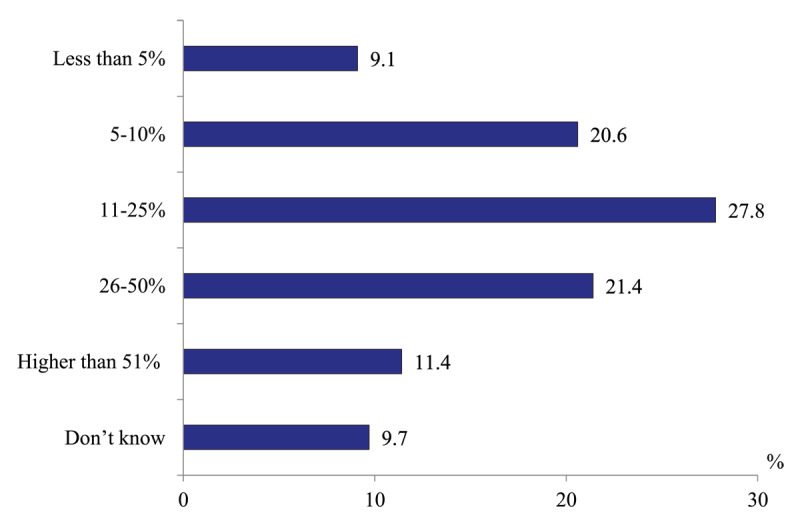
Distribution of polyclinics physicians’ responses to the question “What is your estimate of the share of primary visits to district physicians is finished with the referral to outpatient specialists?” in 2020, %.

*Comprehensiveness of care provided by district physicians*. Only 29.7% of respondents of the survey report that district physicians refer less than 10% of primary patients. 60.6% of our respondents urge that the frequency of referrals is much higher: 27.8% – from 11 to 25% of patients, 32.8 % – more than 25% patients (Figure 13).

**Figure 14:**
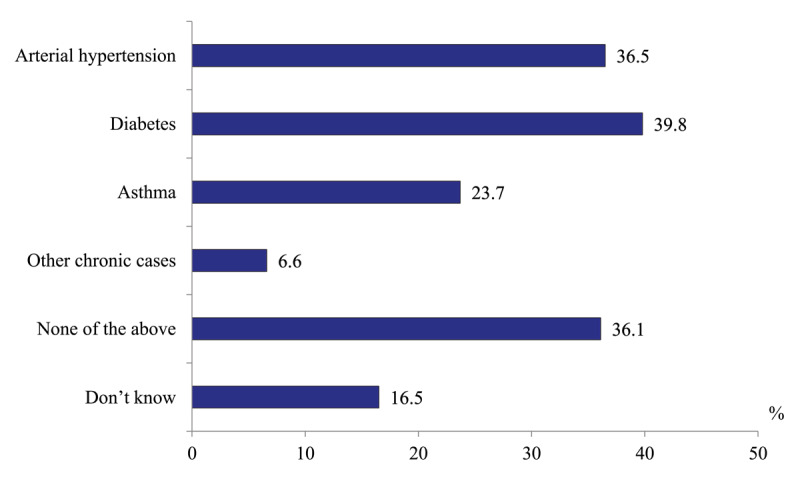
Distribution of polyclinics physicians’ responses to the question “Are there any schools of patients in your polyclinic?” in 2020, %.

*Availability of chronic disease management activities*. We set the question “Are schools of chronic patients organized in your polyclinic?” and addressed it to polyclinic physicians. 36.1% of respondents reported negatively and 16.5% – “don’t know”, while nearly half confirmed the availability of such activities (Figure 14). The questions related to the patients’ coverage, a set of specific services and health outcomes of these schools remained without answer.

**Figure 15:**
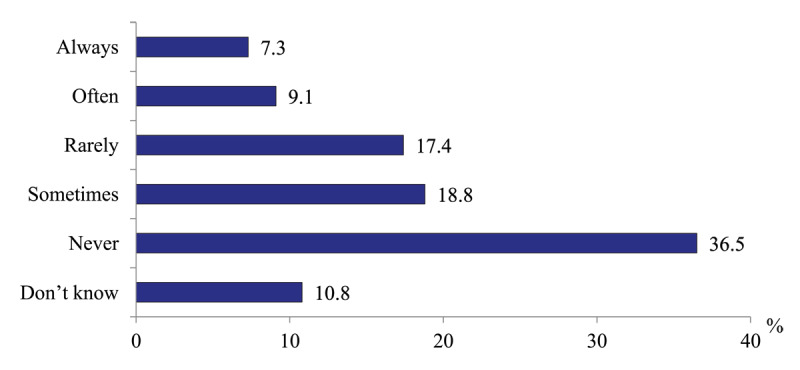
Distribution of polyclinics physicians’ responses to the question “Do you approach social care providers with the request to help your patients?” in 2020, %.

*Initiatives of PHC physicians to involve social workers to the management of patients with substantial health needs*. To determine the degree of such activities, we asked a question “Do you approach social care providers with the request to help your patients?” and addressed it to policlinic physicians. 16.4% report that such initiatives happen “often”, 36.2% – “sometimes” and “rarely”, while every third physician (36.5%) never approaching social workers. Probably, 10.8% of respondents who have problems with the answer are close to the latter (Figure 15).

## Discussion

The study demonstrated that physician survey can add to the evaluation of continuity of care. It differs from those in the above mentioned OECD study [12] and Commonwealth Fund online survey [13]. Following are the major distinctions:

- Our indicators of continuity are focused on the characteristics rather than outcomes of continuity of care, like mortality and readmissions. We explored the gaps in the movement of information and patients in the health system by asking questions about the specific areas of communication between providers. Our list of questions is much longer than in other physician surveys.
- Contrary to other studies, our questionnaire is designed to characterize the types of continuity – informational, longitudinal and interpersonal. This is important to understand specific interactions in the health system.
- We address questions to the predefined categories of physicians (those working in outpatient and inpatient settings) who can professionally make estimates of their specific areas of interactions with other providers. Collecting responses of the “average” physician may distort the responses to many questions.
- Most of our questions are not disease-specific, while the OECD study is focused on two specific health problems [12]. The latter approach is preferable. But it can provide robust estimates only when reflects specific pathways of patient progress in the system. A broad question about the availability of prescribed medicines for post-discharge care is not enough to characterize care continuity.

The limitations of our study lie in its design. A small online survey with the pre-determined minimum number of respondents does not allow to ensure a high representativeness of the sample. It is skewed to young and urban respondents who are more interested in the problem and more active as respondents. The biggest cities of Russia are over-represented, while the structure of respondents across types of care represents the actual structure of physicians in the country. To cope with the limitations of small-scale online survey is difficult. The major instrument is to monitor the intermediate stages of the survey and then repeat invitations to under-represented categories of physicians.

The list of continuity characteristics is not exhaustive. Other characteristics can be added. For the longitudinal continuity, it is possible to explore the following:

– clarity of patients “routes” in the multi-level system of service delivery: are providers aware of the most appropriate next step in the chain of services?
– availability of care coordinator. This is an indication of the attempt to avoid problems at the “junctions” between individual providers and bring together multiple episodes of patient management;
– availability of barriers between public and private providers. They may exist in the countries with the dominance of state owned medical facilities and the variance in the regulation of public and private providers. For example, when there is no requirement of the feedback of private specialists to generalists in public facilities, which is the case in Russia.

For the analysis of interpersonal continuity, GPs’ awareness of their patients’ current health status (particularly patients with complex health needs) can be evaluated. It is also important to evaluate care continuity for vulnerable groups (long-term care residents, people suffering from mental health conditions, etc). The analysis of longitudinal continuity can be strengthened by the evaluation of long-term patient trajectories in the system, for example, the movement of patients with oncological diseases from the moment of initial diagnosis to the start of treatment process. To make a study more comprehensive, a more detailed survey of physicians is needed.

The attempt to determine the role of polyclinic physicians in the management of patients with stroke and heart attack the first days after discharge was not totally successful: most of respondents could not make the estimate of the availability of outpatient care in two weeks time after hospital discharge. Thus the frequency of the follow-up outpatient treatment of such patients remains unclear. With all these limitations, a small online survey provided the opportunity to test the questionnaire and receive some preliminary results. It indicates that the level of care continuity in Russia is low. Access to electronic medical records is limited. Most physicians can share information electronically only with other units of their facility and don’t have a comprehensive picture of health care utilization by their patients. Outpatient and inpatient physicians rarely contact with each other. Polyclinics physicians are unaware of the substantial part of hospital admissions and emergency visits of their patients, which makes them unprepared for the follow-up treatment. Home visits to patients with heart attack and stroke after hospital discharge are rare. The lack of timely transfer of hospital cases to rehabilitative and social care settings also limits continuity of care. The low comprehensiveness of care provided by DPs is a substantial barrier to interpersonal contacts with patients. These findings need to be tested with the use of more representative sample.

The survey based on the specific questions on care continuity can be used for the international comparisons. Following are the major aspects of our survey that can contribute to such comparisons:

– division into types of continuity;
– determining characteristics for each type;
– avoiding broad indicators;
– using most of our questions, with the exclusion of the country-specific questions, for example, the question about feedback of spas to polyclinics;
– addressing questions to the predefined categories of physicians;
– developing uniform requirements to the size of the sample and representativeness of respondents groups across countries.

## Conclusion

The physician online survey can provide additional information on the continuity of care in Russia. It revealed the important zones of fragmentation in the health system that was originally designed as integrated. The survey was designed to increase the number of characteristics and indicators for the evaluation and to make estimates of informational, longitudinal and interpersonal continuity, including specific areas of provider interactions in the health system. The content of the suggested survey can be valuable for collecting an international evidence of continuity and international comparisons. However, a small scale of the survey and its online operation limit its representativeness and robustness. Special activities are needed to overcome the limitations. Bigger scale of the survey with the same or similar questionnaire can improve its results.

## Additional File

The additional file for this article can be found as follows:

10.5334/ijic.7018.s1 Appendix. Physicians’ surveys in 2020 in Russia: distribution of responses.
