# Supplementary material for: Evaluation of Continuity of Care: What Can Physician Survey Add?
Source: Int J Integr Care. 2024 Feb 1;24(1):5. doi: 10.5334/ijic.7018 (PMC10836178; doi:10.5334/ijic.7018)
Supplement: Appendix. — Physicians’ surveys in 2020 in Russia: distribution of responses. [file ijic-24-1-7018-s1.pdf]

Appendix
Physicians' surveys in 2020 in Russia: distribution of responses

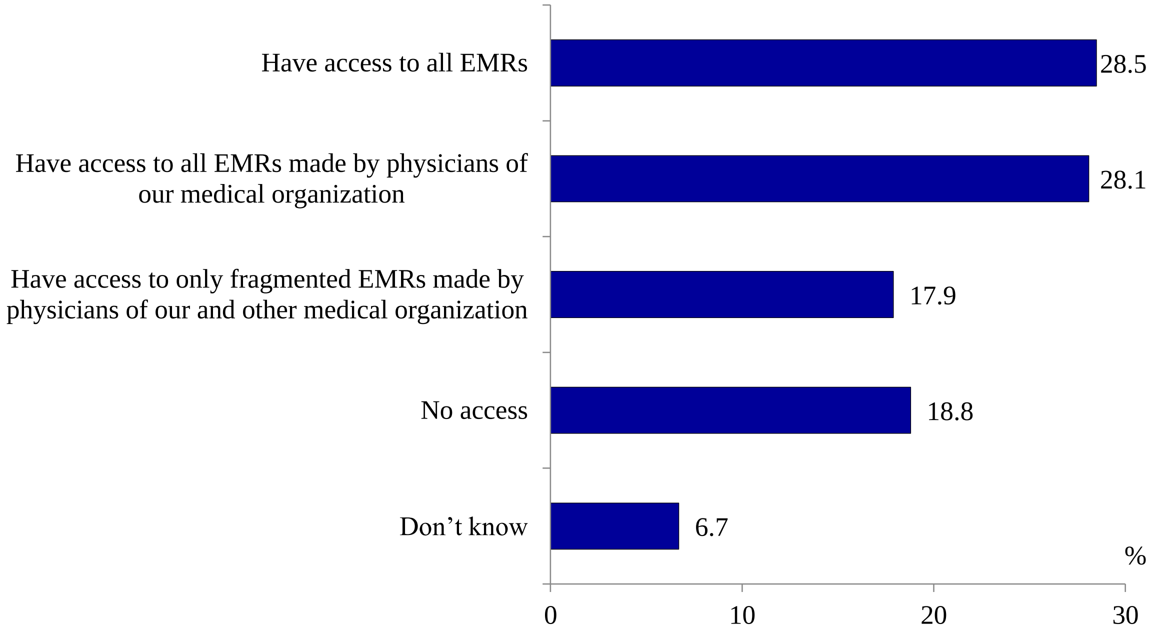

Figure 1. Distribution of polyclinics physicians' responses to the question "Do you have an access to electronic medical records of patients (tests, consultations, admissions, visits, clinical data), if they were made in your region" in 2020, %

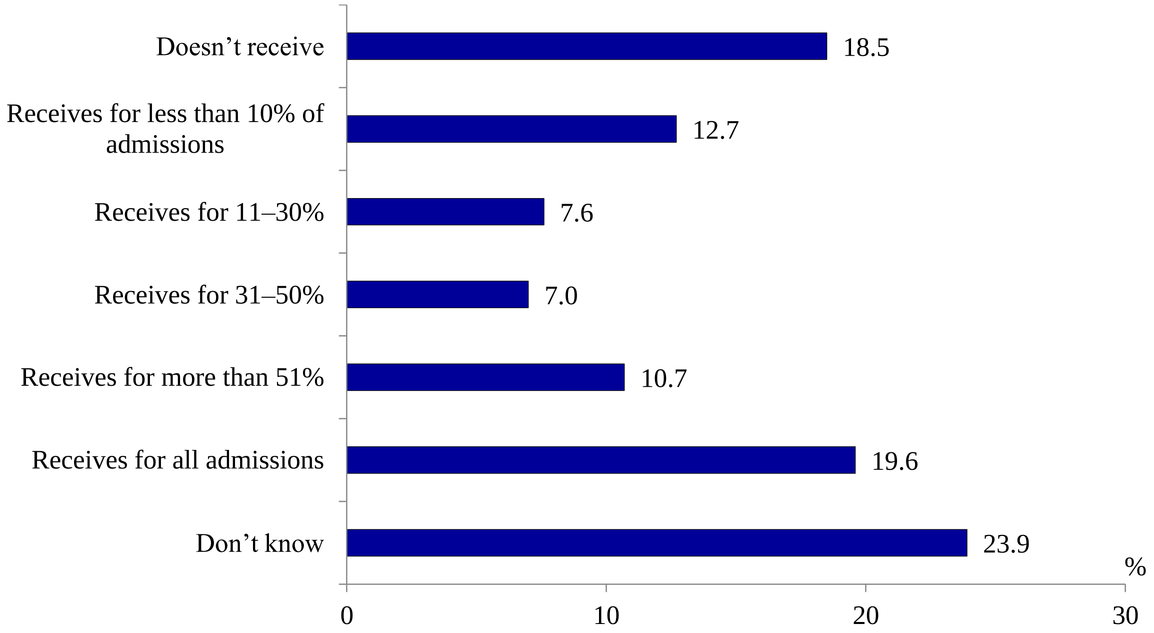

Figure 2. Distribution of polyclinics physicians' responses to the question "How often does your polyclinic receive information about hospital admissions of patients enrolled with it" in 2020, %

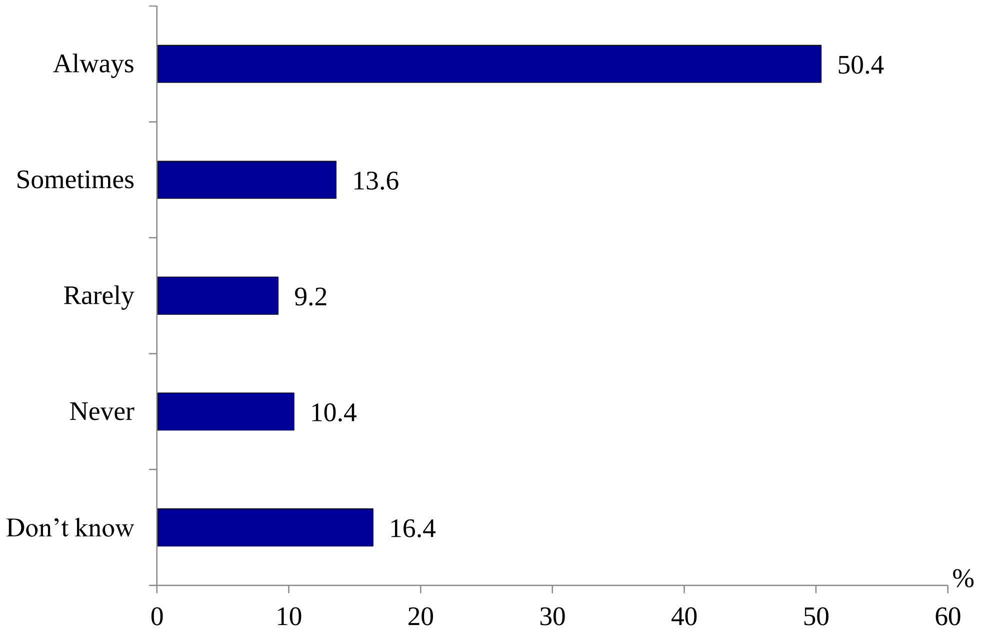

Figure 3. Distribution of polyclinics physicians’ responses to the question “How often does your polyclinic receive information about emergency visits of patients enrolled with it?” in 2020, %

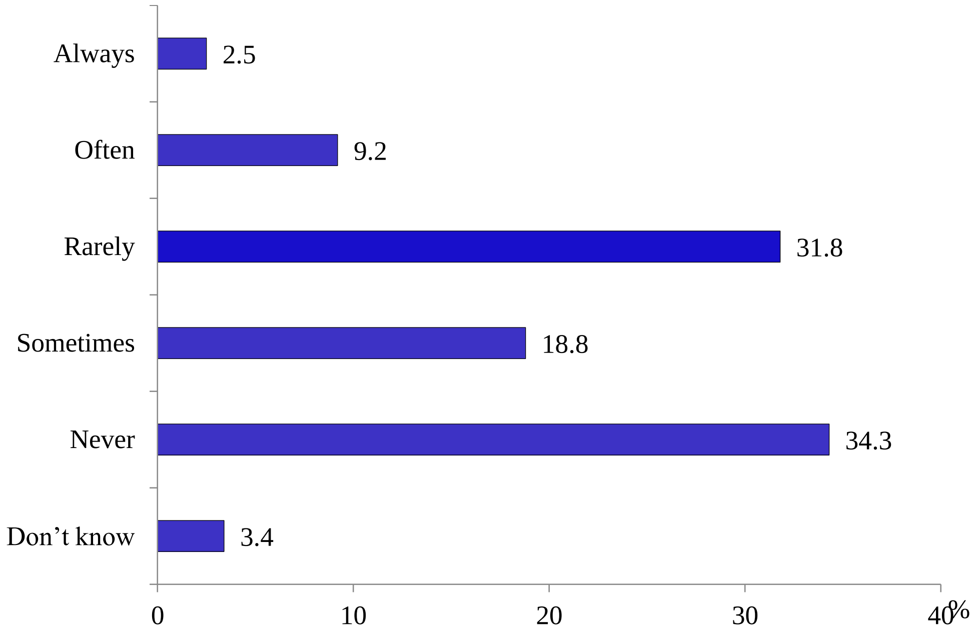

Figure 4. Distribution of hospital physicians’ responses to the question “Do you contact polyclinics physicians when their patients are admitted and treated in a hospital?” in 2020, %

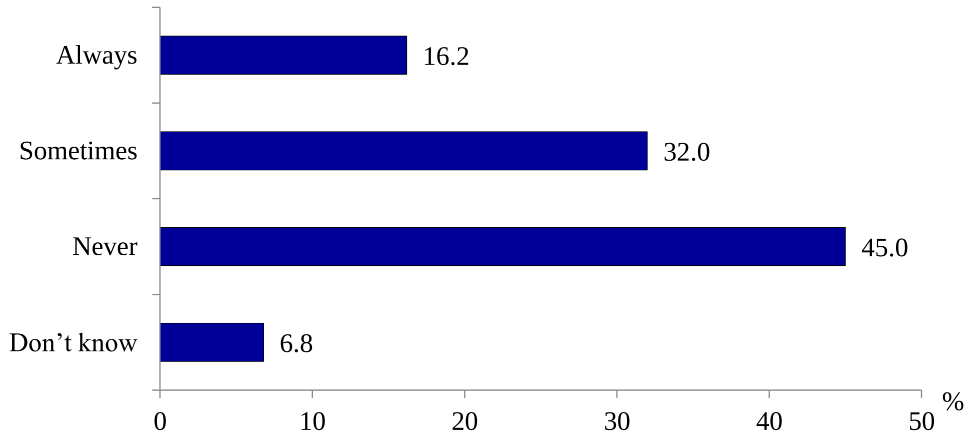

Figure 5. Distribution of polyclinics physicians’ responses to the question “Do you contact hospital physicians about clarifications of post-discharge treatment?” in 2020, %

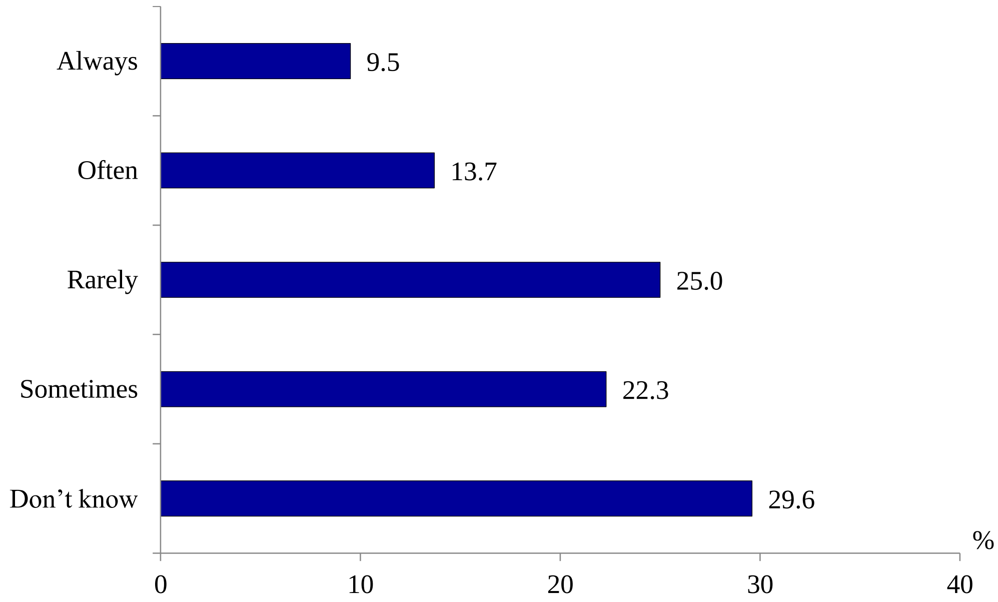

Figure 6. Distribution of polyclinics physicians’ responses to the question “How often do you receive information about results of care in the spa of patients whom you referred?” in 2020, %

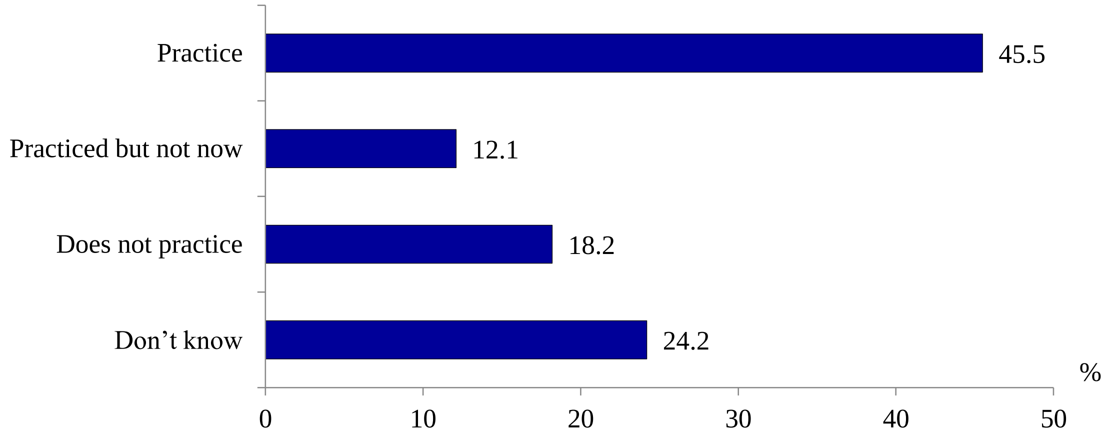

Figure 7. Distribution of polyclinics physicians’ responses to the question “Does your polyclinic practice home visits to patients with stroke and heart attack the first days after their hospital discharge?” in 2020, %

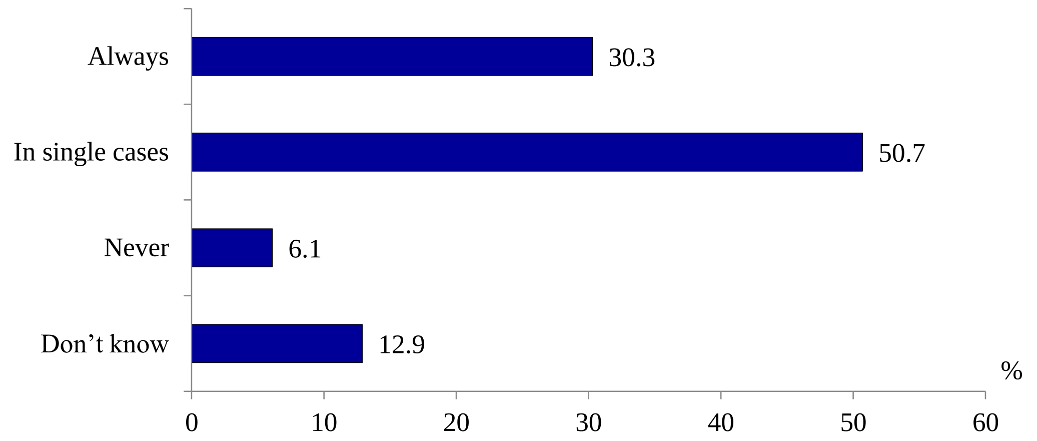

Figure 8. Distribution of hospital physicians’ responses to the question “How often are your patients resubmitted due to inappropriate management by polyclinics physicians?” in 2020, %

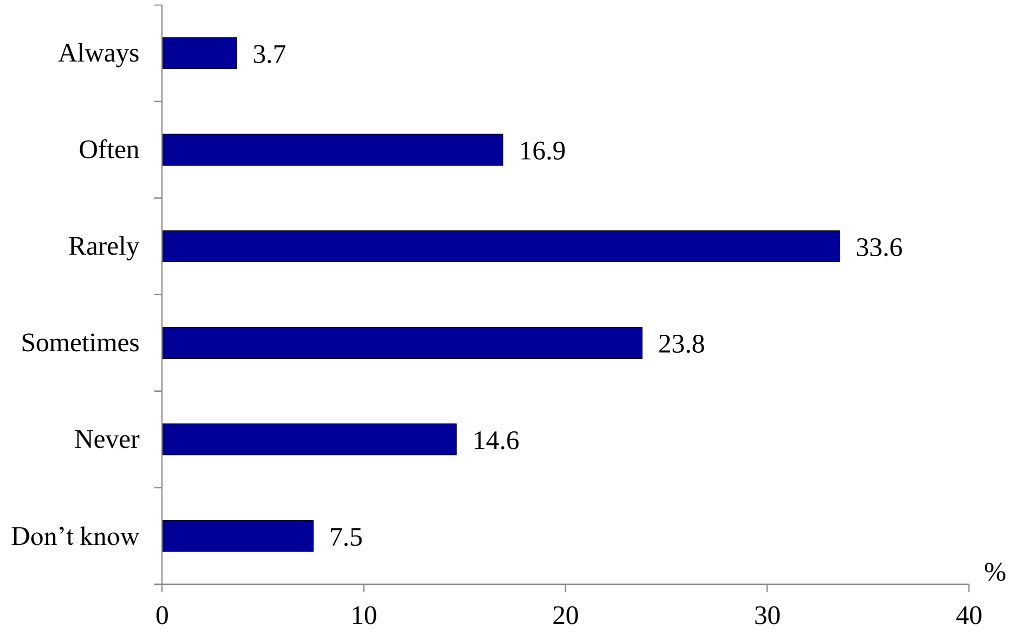

Figure 9. Distribution of hospital physicians’ responses to the question “How often are patients of your hospital transferred to rehabilitative inpatient care entities or units for the continuation of inpatient care (when it is necessary for a patient)?” in 2020, %

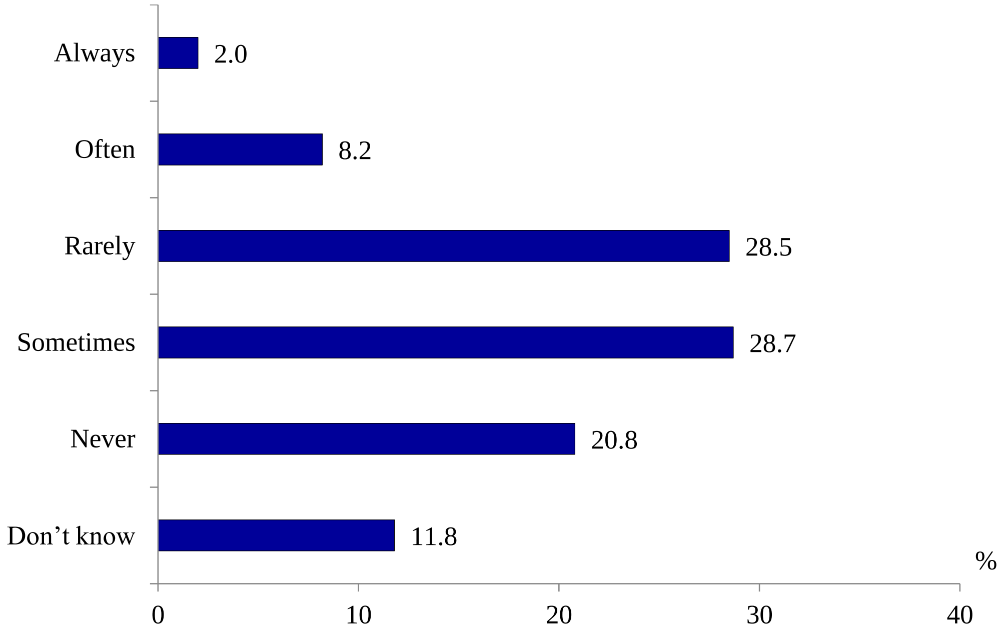

Figure 10. Distribution of hospital physicians’ responses to the question “How often are patients of your hospital transferred to social care entities or units for the continuation of inpatient care (when it is necessary for a patient)?” in 2020, %

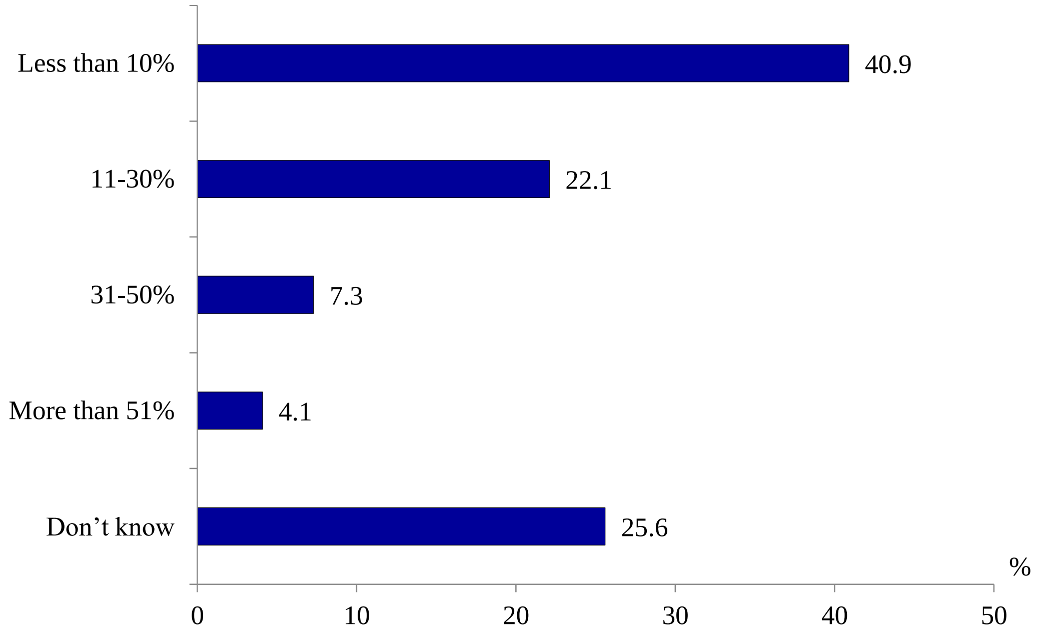

Figure 11. Distribution of hospital physicians’ responses to the question “What is your estimate of the share of hospital beds occupied by patients who need a transfer to the rehabilitative and social entities?” in 2020, %

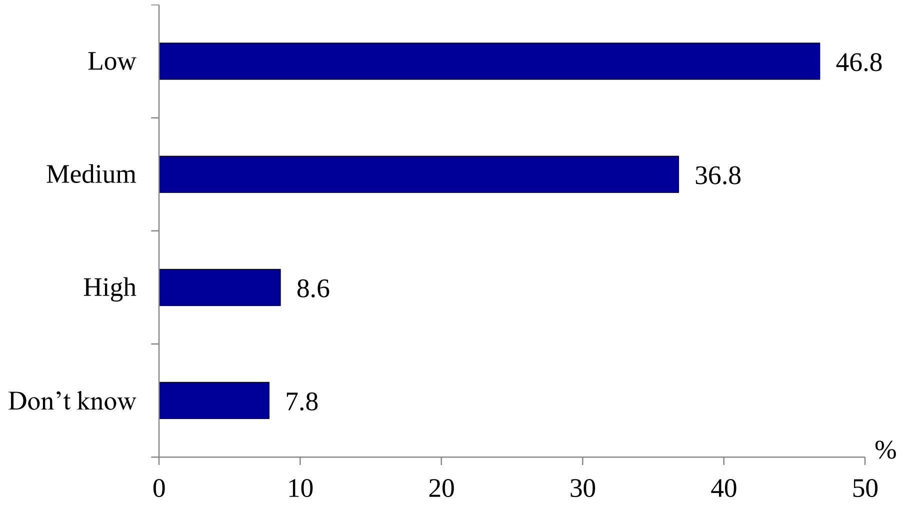

Figure 12. Distribution of all physicians’ responses to the question “What is your estimate of the degree of continuity of care provided in hospitals and polyclinics?” in 2020, %

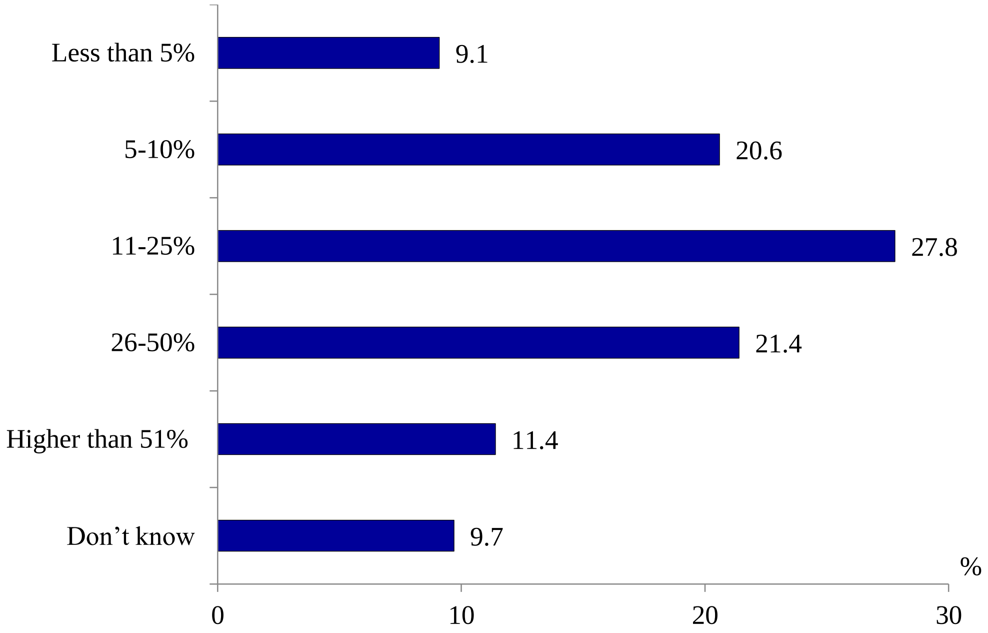

Figure 13. Distribution of polyclinics physicians’ responses to the question “What is your estimate of the share of primary visits to district physicians is finished with the referral to outpatient specialists?” in 2020, %

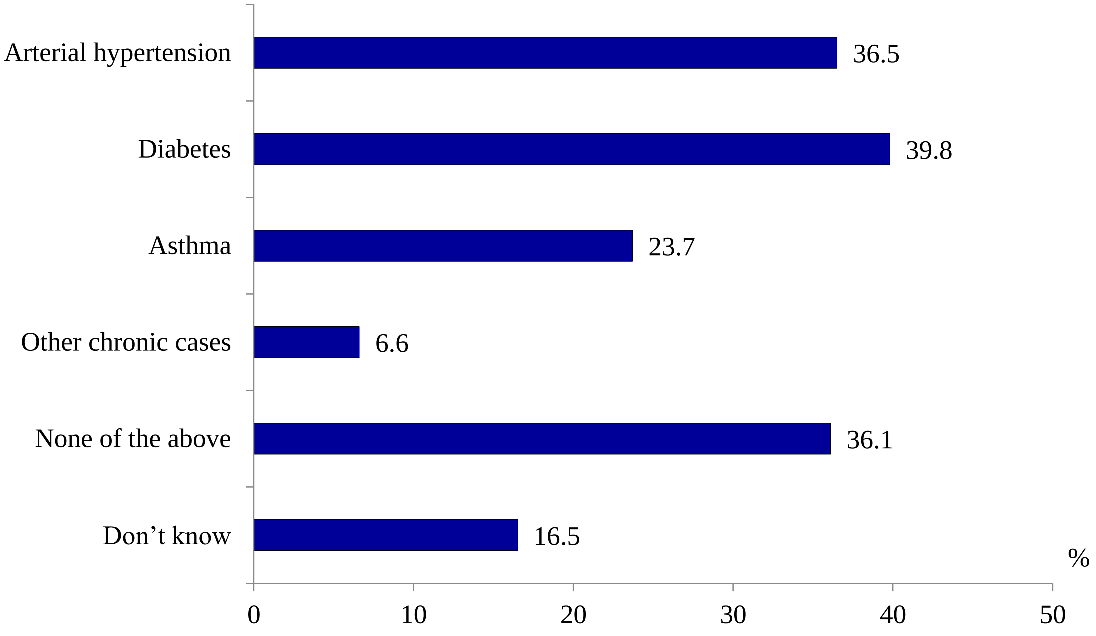

Figure 14. Distribution of polyclinics physicians’ responses to the question “Are there any schools of patients in your polyclinic?” in 2020, %

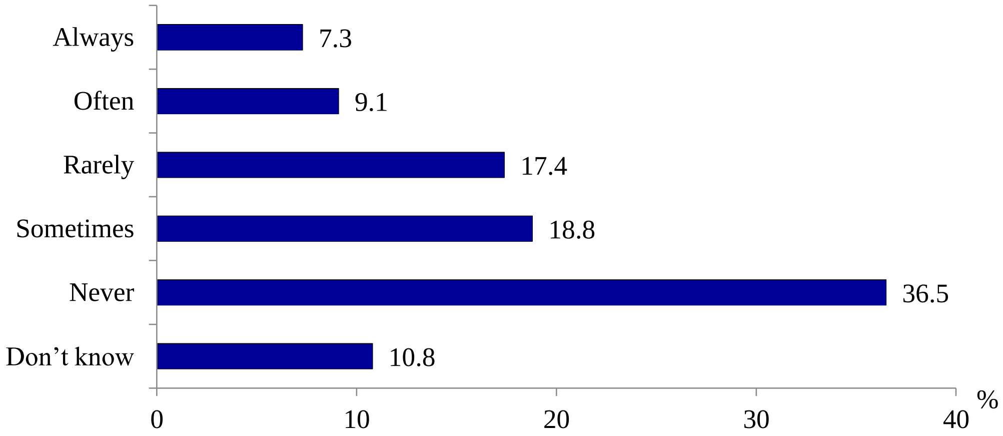

Figure 15. Distribution of polyclinics physicians’ responses to the question “Do you approach social care providers with the request to help your patients?” in 2020, %
